# Do medical conditions predispose to the development of chronic back pain? A longitudinal co-twin control study of middle-aged males with 11-year follow-up

**DOI:** 10.1186/s12891-018-2282-5

**Published:** 2018-10-10

**Authors:** Pradeep Suri, Edward J. Boyko, Sean D. Rundell, Nicholas L. Smith, Jack Goldberg

**Affiliations:** 1 Seattle Epidemiologic Research and Information Center (ERIC), Department of Veterans Affairs Office of Research and Development, Seattle, Washington USA; 2 Division of Rehabilitation Care Services, VA Puget Sound Health Care System, 1660 S. Columbian Way, Seattle, WA 98108 USA; 3 Department of Rehabilitation Medicine, University of Washington, Seattle, Washington USA; 4 General Medicine Service, VA Puget Sound Health Care System, Seattle, Washington USA; 5 Department of Epidemiology, University of Washington, Seattle, Washington USA; 6 Kaiser Permanente Washington Research Institute, Kaiser Permanente Washington, Seattle, Washington USA

**Keywords:** Twins, Coronary artery disease, Atherosclerosis, Diabetes, Hypertension, Musculoskeletal diseases

## Abstract

**Background:**

Poor general health predicts the transition to chronic back pain (CBP), but the role of specific medical conditions in the development of CBP is unclear. The study aim was to examine the association of medical conditions with the development of CBP (“incident CBP”), while controlling for familial factors, including genetics.

**Methods:**

This was a longitudinal co-twin control study conducted in a nationwide United States sample from the Vietnam Era Twin Registry. The study sample included 3045 males without back problems at baseline, including 662 complete twin pairs, who were followed for 11 years. Baseline surveys inquired about self-reported medical conditions (arthritis, diabetes, hypertension, and coronary artery disease [CAD]). A medical comorbidity score was calculated based on the presence and/or treatment of 8 medical conditions. Covariates included age, race, and education. At 11-year follow-up, participants reported ever having had CBP. Odds ratios (ORs) and 95% confidence intervals (CI) were estimated when considering twins as individuals, and in matched-pair co-twin control analyses adjusting for familial/genetic factors.

**Results:**

Mean age at baseline was 51 years and 17% of participants developed CBP over the 11-year follow-up. Arthritis was significantly associated with incident CBP in individual-level analysis (OR 1.8 [95% CI 1.4–2.2]), but not within-pair analysis (OR 0.9 [95% CI 0.4–1.9]. CAD (OR 1.6 [95% CI 1.0–2.3]), hypertension (OR 1.3 [95% CI 1.0–1.5]), and the medical comorbidity score (OR 1.2 [95%CI 1.1–2.2]) were significantly associated with incident CBP in individual-level analyses; associations in within-pair analyses were of comparable magnitude, but not statistically significant. Diabetes was not associated with incident CBP.

**Conclusions:**

Arthritis, hypertension, CAD, and medical comorbidity score were associated with incident CBP in the current study. However, the association between arthritis and incident CBP was confounded by familial factors. This suggests that prevention or treatment of arthritis is unlikely to be useful for CBP prevention. Our findings cannot exclude the possibility of causal associations between CAD, hypertension, and medical comorbidities and incident CBP.

**Electronic supplementary material:**

The online version of this article (10.1186/s12891-018-2282-5) contains supplementary material, which is available to authorized users.

## Background

The symptom of back pain affects most adults at some point in their lives [1], and causes more years lived with disability than any other health condition worldwide [2]. The societal burden of back pain is driven by the minority of individuals who do not recover from a new back pain episode, and who go on to develop ‘chronic’ back pain (CBP) [3]. Accordingly, much research has attempted to identify preventable conditions associated with the development of CBP [4].

CBP is recognized as a complex condition best studied and managed within the context of a biopsychosocial framework, as opposed to a strictly biomedical model. Psychological factors such as depression, anxiety, fear-avoidance, catastrophizing, and self-efficacy have been extensively studied as risk factors for CBP [5]. Much effort has also been expended to identify specific spine-related conditions linked to CBP [6–9]. Fewer studies, however, have examined the role of medical conditions not involving the spine as risk factors for CBP. Poor general health is a known risk factor for new (‘acute’) back pain [10] and the acute-to-chronic back pain transition [4]. Specific medical conditions might have particular importance as risk factors for CBP, especially if such conditions have a causal role in CBP, and if they can be prevented or treated. Self-reported and clinically diagnosed arthritis and joint problems predict CBP and poor back-related outcomes [11–14]. Several proposed explanations for the link between arthritis and CBP include that lower limb arthritis leads to postural or biomechanical changes which place increased stresses on the back [15]; that pain in the back may originate from or reflect progression of arthritic structures external to the spine (e.g. the hip joint) [16]; that an underlying propensity to generalized arthritis predisposes to future arthritic involvement of the spinal structures and consequent back pain; that self-reported ‘arthritis’ simply reflects an underlying susceptibility to painful conditions such as back pain; and semantic issues whereby some individuals do not distinguish between the terms ‘arthritis’ and ‘back pain’ [15]. In addition, cardiovascular risk factors such as diabetes and hypertension have been implicated in back pain and spinal disorders through putative mechanisms involving lumbar arterial atherosclerosis [17, 18]. Atherosclerotic lesions in branching arteries supplying the lumbar spine may cause impaired nutrition to the vertebrae, intervertebral discs, and nerve roots, leading to disc degeneration and consequent back pain [18–22]. However, it is unclear whether any of these medical conditions actually confer a greater risk of CBP, or whether they are associated with CBP due to other reasons. For instance, individual medical conditions may simply serve as proxies for poor general health, an idea supported by a recent study demonstrating that the number of self-reported conditions predicted future back pain in men [23]. Much of the research supporting a relationship between medical conditions and CBP consists of cross-sectional studies, which cannot identify temporal sequence, and are particularly prone to confounding by other factors [15, 17, 24–29]. Although there are many possible sources of confounding which might underlie the link between medical conditions and CBP, one important explanation is shared underlying vulnerabilities, either genetic or familial, which predispose to both medical conditions and CBP. Observational study designs using genetically informative samples may be used to examine associations between medical conditions and CBP free of confounding by these shared underlying vulnerabilities.

The aim of this study was to examine the association of self-reported medical conditions with the development of CBP, using a genetically informative longitudinal co-twin control study design to account for confounding due to familial factors, including genetics. Based on prior literature, the self-reported medication conditions examined in this study included arthritis and cardiovascular risk factors/conditions (diabetes, hypertension, and coronary artery disease [CAD]).

## Methods

### Overview of the co-twin control design

A major challenge for observational research in CBP is how to isolate the effects of specific risk factors by controlling for relevant confounding factors. Most commonly this is done by multivariate statistical adjustment, yet even with the most rigorous methods there remains the potential for residual confounding due to unknown or unmeasured factors. An alternative approach is to design studies that may account for some of the unknown or unmeasured confounding factors. One such approach is to use the *co-twin control design* to examine the relationship between a putative risk factor and CBP. Contemporary approaches to the co-twin control design permit comparing the association of a risk factor and CBP using both ‘individual-level’ analyses (such as those used in conventional studies of unrelated individuals), with ‘within-pair’ analyses comparing twins to their co-twins [30]. Within-pair analyses account for ‘familial factors’, including both genetic factors and early life experiences (also called ‘shared’ environmental factors), which are common to both twins. Such within-pair analyses are matched for factors such as age and ethnicity, as well as other factors which are not directly measured, but which twins share. Since genetic predispositions and early life experiences which influence different health conditions are accounted for in the within-pair analyses, associations between putative risk factors and CBP which persist in within-pair analyses are less likely to be explained by confounding due to genetic or shared environmental factors, and might be possibly modifiable and therefore targets for intervention [31].

### Study participants

This longitudinal co-twin control study was a secondary analysis of existing data from the Vietnam Era Twin (VET) Registry. The VET Registry was constructed from military discharge records, and is a national sample of male twin pairs from all United States (US) military branches who were born between 1939 and 1957 and who served on active duty during the Vietnam era (1965–1975) [32]. The VET Registry was not compiled based on specific diagnoses, health behaviors, or military service characteristics aside from military discharge; details of the Registry construction and zygosity ascertainment process have been described previously [33–35]. VET Registry members reside in all 50 US states, and are comparable to older US males from the general population with respect to income and education; most obtain healthcare outside the Veterans Affairs (VA) system [36].

**Fig. 1:**
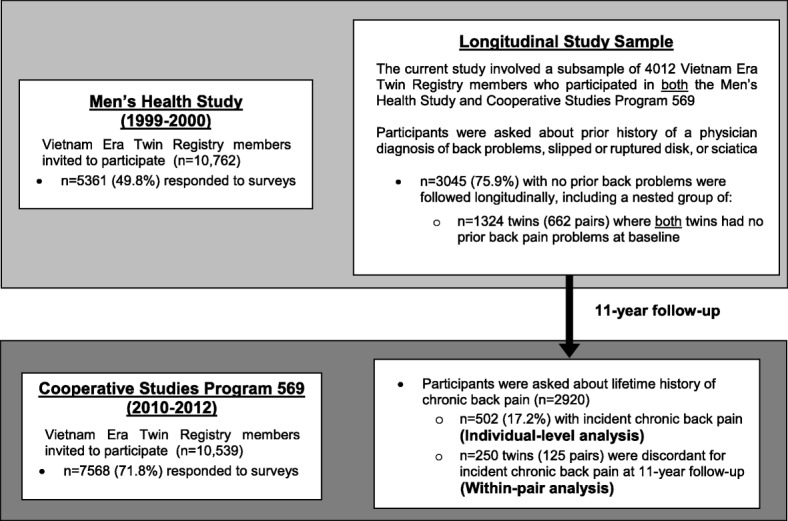
Flowchart of Study Participation

In 1999–2000, VET Registry members were invited to participate in an observational study of men’s health, the ‘Men’s Health Study’, which included a mailed survey obtaining information regarding self-reported medical conditions and health behaviors. Responses from this survey formed the baseline assessment for the current analysis. Between 2010 and 2012, VET Registry members were invited to participate in an observational study of PTSD among Veterans (VA Cooperative Studies Program #569: The Course and Consequences of PTSD in Vietnam Era Twins, or ‘CSP #569’). This included a mailed survey obtaining information about mental and physical health conditions. The current analysis included VET Registry participants who participated in both the Men’s Health Study and CSP#569, 11 years later (Fig. 1). Written informed consent was obtained from all VET Registry members, and study procedures were approved by the VA Puget Sound Institutional Review Board.

### Assessment of medical conditions

The main medical conditions of interest for the current study measured at study baseline during the Men’s Health Study were arthritis, diabetes, hypertension, and CAD (which was considered a proxy for general atherosclerosis [19–22]). At the baseline assessment, participants reported whether they had ever previously been diagnosed with specific medical conditions, choosing from a list of common conditions, including “arthritis of any kind, or rheumatism”, “diabetes”, “hypertension or high blood pressure”, “coronary heart disease”, “asthma”, “chronic bronchitis”, “emphysema or chronic obstructive pulmonary disease”, “gastroesophageal reflux disease or reflux esophagitis”, “kidney disease”, or “liver disease”. For each condition, participants also reported whether they had received medical treatment for the condition in the past year. Reported medical conditions were not independently corroborated by other means. For the purposes of the current study, the definitions of the main medical conditions of interest were: arthritis defined as self-report of prior diagnosis of “arthritis of any kind, or rheumatism”, without specification as to the type of arthritis, or the joints involved; diabetes defined as self-report of prior diagnosis of “diabetes”, without distinguishing type 1 diabetes from type 2 diabetes; hypertension defined as self-report of “hypertension or high blood pressure”; and CAD defined as self-report of “coronary heart disease”.

Data on self-reported medical conditions were used to generate an overall score reflecting the burden of major medical conditions (the ‘comorbidity score) in a manner analogous to that used in the Self-Administered Comorbidity Questionnaire (SACQ), a validated self-report measure for comorbidity burden that is commonly used in orthopedic research [37]. The comorbidity score used these medical diagnosis groups included in the SACQ: heart disease, hypertension, lung disease, diabetes, ulcer/stomach disease, kidney disease, liver disease, and arthritis. For calculation of the comorbidity score, lung disease was defined as a self-report of “asthma”, “chronic bronchitis”, or “emphysema or chronic obstructive pulmonary disease”, and ulcer/stomach disease was defined as “gastroesophageal reflux disease or reflux esophagitis”. Although typically part of the SACQ, back pain was not included in our comorbidity score since it was the outcome of interest, and information for three other diagnosis groups from the SACQ (anemia, cancer, and depression) were also not available as part of the baseline assessment in the Men’s Health Study. Scoring followed the same general practice as used in the SACQ: individuals could receive up to a maximum of 2 comorbidity ‘points’ for each medical condition: 1 point for the presence of the condition, and another point for having received treatment for it in the past year. The number of points were summed across all 8 conditions, for a possible range between 0 (lowest possible comorbidity burden) to 16 (highest possible burden). This comorbidity score differed from the SACQ in that the SACQ also includes a 3rd item inquiring whether each condition results in functional limitations, permitting up to 3 comorbidity points for each condition [37].

### Development of Chronic back pain

At baseline, respondents reported whether or not they had ever previously been told by a doctor that they had ‘back problems’, slipped or ruptured disk, or sciatica. Individuals who reported having no prior back problems at baseline constituted the study sample that was followed longitudinally. At the follow-up assessment 11 years later, respondents reported whether they had ever had chronic back pain, without specification as to the location or duration of their back pain. Those participants without back problems at the study baseline, who went on to report chronic back pain 11 years later, were classified as having developed CBP (‘incident CBP’).

### Covariates

Adjustment variables and potential confounders included participant age, race, and educational attainment. Data obtained from military records classified race as white vs. non-white. Participants reported educational attainment as the highest grade/year of school completed, and the highest degrees obtained.

### Statistical analysis and interpretation

**Fig. 2:**
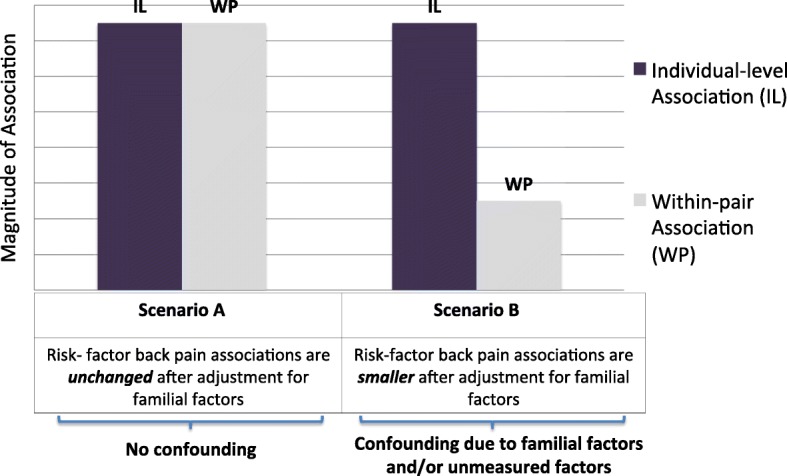
Possible Scenarios of Confounding of Risk Factor-Back Pain Associations due to Familial Factors*. *The term ‘familial factors’ includes both genetic factors and early family environmental factors

We used statistical methods for co-twin control studies [31] to examine associations between baseline medical conditions and incident CBP in longitudinal analyses restricted to individuals who did not report back problems at baseline. First, we conducted multivariable-adjusted individual-level analyses equivalent to that used in conventional studies of unrelated individuals, using generalized estimating equations (GEE) to account for clustering by twin pair when calculating odds ratios (ORs) and 95% confidence intervals (CIs). In these analyses we adjusted for age, race, and education, yielding effect estimates similar to those yielded by any conventional epidemiology study. Due to the interrelatedness of the different cardiovascular factors examined, and to avoid conditioning on intermediates along theoretical causal pathways, only one medical condition at a time was included in the multivariate models [38]. In a separate analytic step of the individual-level analysis, we also included the comorbidity score as an additional adjustment variable. In each such model, the main medical condition of interest was excluded from calculation of the comorbidity score so that medical conditions were not ‘counted’ twice; for instance, the model for the arthritis-incident back pain association adjusted for the comorbidity score, but for the purpose of that specific model, arthritis was not included in calculation of the comorbidity score. Next, we conducted within-pair analyses comparing twins to their co-twins using conditional logistic regression analyses, in which only twin pairs who are discordant for the CBP outcome are informative. These within-pair analyses account for familial factors (which include both genetic and early ‘shared’ family environmental factors) as well as unmeasured confounders due to the similarities within a twin pair. The degree to which within-pair estimates differ from individual-level estimates of association can be used to infer whether familial factors are a source of confounding [31]. If individual-level and within-pair associations are of similar magnitude, this implies no confounding or minimal confounding due to familial factors (Fig. 2, Scenario A). If, however, familial factors confound the association between a health-related factor and CBP then the magnitude of association in within-pair analyses will typically be closer to the null (ie, an OR of 1.0) than that observed in the individual-level analyses (Fig. 2, Scenario B) [39]. The analytic approach involved complete-case analysis. Small sample sizes are common in longitudinal within-twin-pair analyses using categorical outcomes, since only twin pairs where both twins lack the outcome at baseline, and where twins are discordant for the outcome at follow-up, are informative. Thus, inferences regarding the strength of associations must not only refer to statistical significance, but also to the magnitude of point estimates and width of confidence intervals (CIs). Due to sample size concerns, the within-pair analyses were not stratified by twin zygosity (monozygotic vs. dizygotic); however, for completeness, zygosity-stratified results are provided in Additional file 1.

## Results

Among 3045 participants who reported no prior history of back problems at baseline (Fig. 1), the mean age was 50.5 years. The cumulative incidence of CBP over 11-year follow-up was 17.2% (95% CI 15.9–18.6). Those with incident CBP were younger, and had lower levels of education compared with participants without incident CBP (Table 1). Participants with incident CBP were significantly more likely than those without to report arthritis, hypertension, and CAD, and have a higher medical comorbidity score. From among the longitudinal sample of 2920 participants, there were only 250 twins (125 pairs) without back problems at baseline and who were discordant for incident CBP over 11-year follow-up and thus informative in the within-pair analyses.

**Table 1:** Characteristics of the Longitudinal Study Sample*

Characteristics	Incident Chronic Back Pain Over 11-Year Follow-up		
	No (*n* = 2418)	Yes (*n* = 502)	*p* value*
Sociodemographics			
Age (mean ± standard deviation)	50.6 ± 3.0	50.0 ± 3.1	< 0.001
Race (%)			
White	2322 (96.3%)	481 (96.0%)	0.75
Non-white	90 (3.7%)	20 (4.0%)	
Educational attainment			
Did not graduate high school	55 (2.4%)	22 (4.6%)	0.01
High school graduate	494 (21.4%)	110 (23.1%)	
Some college/vocational school^a^	1016 (44.0%)	215 (45.2%)	
Completed college or further study^b^	744 (32.2%)	129 (27.1%)	
Medical Comorbidities			
Arthritis	331 (13.7%)	111 (22.2%)	< 0.001
Diabetes	112 (4.6%)	30 (6.0%)	0.22
Hypertension	508 (21.1%)	127 (25.5%)	0.03
Coronary Artery Disease (CAD)	95 (3.9%)	30 (6.0%)	0.03
Overall Comorbidity Burden			
Medical comorbidity score, range 0–18 (median [IQR])	0 [0–2]	1 [0–2]	< 0.001

*p-values reflect clustering by twinship

^a^Including some college without completion, or having completed vocational school, technical school, or a 2-year college degree

^b^attended and/or completed graduate school

### Medical conditions and incident CBP over 11-year follow-up

Self-reported arthritis was significantly associated with incident CBP in multivariable-adjusted individual-level analyses (OR 1.8 [95% CI 1.4–2.3]; *p* < 0.001), and further adjustment for comorbidity score resulted in a slight decrease (OR 1.7) in the magnitude of this association (Table 2). However, the association was notably attenuated and no longer significant in the within-pair analysis (OR 0.9 [95% CI 0.4–1.8]; *p* = 0.72).

**Table 2:** Incidence of Chronic Back Pain over 11-year Follow-up

Risk Factor	Individual-level analysis^a^		Individual-level analysis (also adjusting for comorbidity score)^b^		Within-pair analysis	
	OR (95% CI)	*p*-value	OR (95% CI)	*p*-value	OR (95% CI)	*p*-value
Arthritis	*n* = 2770		*n*=2745^c^		*n* = 248 (124 pairs)	
	**1.8 (1.4–2.2)**	**< 0.001**	**1.7 (1.3–2.2)**	**< 0.001**	0.9 (0.4–1.8)	0.72
Diabetes	*n* = 2776		*n *= 2748^d^		*n* = 248 (124 pairs)	
	1.2 (0.8–1.9)	0.33	1.2 (0.8–1.9)	0.46	0.7 (0.2–2.4)	0.53
Hypertension	*n* = 2770		*n *= 2745^d^		*n* = 248 (124 pairs)	
	**1.3 (1.0–1.5)**	**0.04**	1.2 (0.9–1.5)	0.14	1.3 (0.6–2.6)	0.48
Coronary Artery Disease (CAD)	*n* = 2771		*n *= 2744^d^		*n* = 248 (124 pairs)	
	**1.6 (1.0–2.3)**	**0.05**	1.5 (0.9–2.3)	0.09	2.0 (0.5–8.0)	0.33
Overall Comorbidity Burden						
Medical comorbidity score	*n* = 2740		–		*n* = 240 (120 pairs)	
	**1.2 (1.1–2.3)**	**< 0.001**	**–**	–	1.1 (0.9–1.4)	0.32

Associations between medical conditions and incident chronic back pain, in those without physician-assessed back problems at baseline *

Items **in bold** are statistically significant at *p* < 0.05

Sample sizes indicate # of individuals with complete data for these variables, and within-pair analyses are restricted to pairs with complete data for all variables

^a^Models adjusting for age, race, education

^b^Models adjusting for age, race, education, and comorbidity score

^c^Model adjusting for age, race, education, and comorbidity score (arthritis not included in calculation of comorbidity score)

^d^Model adjusting for age, race, education, and comorbidity score (diabetes, hypertension, and CAD not included in calculation of comorbidity score)

Diabetes was not significantly associated with incident CBP in individual-level nor within-pair analyses (Table 2). Hypertension was significantly associated with incident CBP in multivariable-adjusted individual-level analyses (OR 1.3 [95% CI 1.0–1.5]; *p* = 0.04); the magnitude of this association was slightly smaller and not statistically significant after further adjustment for comorbidity score (OR 1.2 [95% CI 0.9–1.5]; *p* = 0.14). Although the magnitude of the hypertension-CBP association was slightly larger in within-pair analysis, the confidence intervals were wider and the results not statistically significant (OR 1.3 [95% CI 0.6–2.6]; *p* = 0.48). CAD was significantly associated with incident CBP in multivariate-adjusted individual-level analyses (OR 1.6 [95% CI 1.0–2.3]; *p* = 0.05)); the magnitude of this association was slightly smaller and not statistically significant after further adjustment for comorbidity score (OR 1.5 [95% CI 0.9–2.3]; *p* = 0.09). Within-pair analyses yielded wide confidence intervals and associations that were not statistically significant, although associations were of comparable magnitude (OR 2.0 [95% CI 0.5–8.0]; *p* = 0.33) to the individual-level analyses. Further adjustment of the within-pair analyses in Table 2 of specific medical conditions for comorbidity score showed no material differences (data not shown).

The medical comorbidity score was significantly associated with incident CBP in multivariate-adjusted individual-level analyses (Table 2) (OR 1.2 per comorbidity point [95% CI 1.1–2.3]; *p* < 0.001). The magnitude of this association was slightly smaller in within-pair analyses (OR 1.1 per comorbidity point [95% CI 0.9–1.4]; *p* = 0.32) and not statistically significant.

Within-pair analyses stratified by zygosity were not materially different from the main within-pair analyses, although confidence intervals generally were wider due to the smaller sample sizes involved (see Additional file 1).

## Discussion

This study found that among medical conditions examined in individual-level analyses, self-reported arthritis, CAD, hypertension, and a medical comorbidity score were significantly associated with incident CBP at 11-year follow-up, consistent with some prior reports. The associations of specific medical conditions with incident CBP were, to a small degree, accounted for by general comorbidity burden as measured by the medical comorbidity score. Co-twin control analyses indicated that the arthritis-CBP association was confounded by familial predispositions underlying both conditions, arguing against a causal link. However, the results of other co-twin control analyses could not exclude the possibility that there is some effect of CAD and hypertension, and medical comorbidities in general, on the development of CBP.

Variables predictive of a health condition are not necessarily causal [40]. Self-reported arthritis and joint problems predict CBP and poor back-related outcomes in clinical studies [11–13]. Although some have proposed a ‘knee-spine syndrome’ whereby lower limb arthritis or joint problems lead to biomechanical alterations in activities such as ambulation, which then lead to back pain [15], many other explanations exist aside from a causal link [29]. Our individual-level analysis results are consistent with prior studies of the arthritis-back pain relationship, showing that self-reported arthritis is associated with a substantially greater likelihood of developing CBP over 11-year follow-up (OR point estimate = 1.8). Thus, self-reported arthritis may have value as a marker for individuals who are more likely to experience CBP in the future. Although adjustment for other medical comorbidities had minimal effect on the arthritis-incident CBP association, there was no meaningful association between arthritis and incident CBP (OR 0.9) in the within-pair analyses that reflect adjustment for familial confounding. Strong inferences cannot be made based on the within-pair analyses due to the small sample sizes and wide confidence intervals involved, however, this overall pattern of results suggests a role of shared genetic factors in the arthritis-CBP relationship, and argues against arthritis being an actual determinant of future CBP. The role of shared genetics in the arthritis-CBP relationship is supported by the recent results of a large-scale genetic association study involving more than 158,000 individuals, which found large-magnitude genetic correlations (0.63) between self-reported CBP and self-reported osteoarthritis [41].

Research has shown that cardiovascular disease and related risk factors (abdominal aortic atherosclerosis in particular) are associated with disc degeneration and back pain [19, 20, 22], prompting speculation that treatment of cardiovascular risk factors/conditions might also help to prevent CBP or minimize its impact [22, 42–44]. Results from our individual-level analyses of hypertension and CAD are consistent with earlier reports that these conditions are associated with future back pain or spine-related symptoms [18, 22]. These associations were slightly smaller when adjusting for the comorbidity score, indicating that a small component of these associations might be due to hypertension and CAD reflecting manifestations of poor general health (and CBP might also reflect another aspect of general health). However, although the within-pair analyses for hypertension and CAD were limited by imprecision, the comparable magnitude estimates of association as compared to the individual-level analyses argue against shared underlying predispositions to cardiovascular risk factors/conditions and back pain as a complete explanation for why cardiovascular factors predict future back pain [42]. These findings leave open the possibility that there is a causal effect of hypertension, CAD, and/or general medical comorbidity on the development of CBP.

Our finding that diabetes is not associated with future CBP largely fits in the context of prior longitudinal studies. One such study of diabetes’ association with future back pain also yielded a null association [45]. However, other studies examining the association of diabetes with the future occurrence of other spine-related phenotypes such as physician-diagnosed lumbar disc herniation and general musculoskeletal pain (including back pain) have found positive associations [22, 44]. Thus, the association of diabetes with musculoskeletal pain may be driven by non-back locations, or may pertain only to certain subsets of people with back pain.

To our knowledge, this is the first longitudinal co-twin control study of back pain in a US sample. Co-twin control studies are often performed to sharpen our understanding of why two phenotypes, such as CBP and arthritis, might be associated. In our application of the co-twin control approach we observed that shared familial factors, including genetic factors, may underlie the association of arthritis and CBP. Support for shared genetic influences on CBP and arthritis come from other recent work by our group: a genome-wide meta-analysis of CBP identified and replicated a variant in the gene *SOX5*, previously implicated in osteoarthritis [46–48], that was a significant predictor of CBP [49]. Future genetic studies may benefit from harnessing knowledge regarding genetic influences on CBP shared with other musculoskeletal phenotypes, such as arthritis. For instance, multivariate genome-wide association studies, in which genetic associations with several traits are analyzed together, have advantages in statistical power over univariate analyses of each trait separately, in some instances [50].

Our study had limitations with regards to the definitions used for the predictor and outcome variables of interest. Similar to most prior longitudinal studies showing relationships between medical conditions and back pain [22, 23, 44], our study relied entirely on self-report. For instance, ‘arthritis’ in the current study may have reflected either osteoarthritis or inflammatory arthritis in the major lower extremity joints (i.e. hip or knee), or the hand joints, or elsewhere. Similarly, ‘diabetes’ in the current study may reflect either type 1 or type 2 diabetes. We expect that these self-report definitions used in our study reflect the influence of the most prevalent underlying conditions, such that the ‘arthritis’ variable mainly reflects the most commonly symptomatic arthritic conditions affecting older adults (knee osteoarthritis, hip osteoarthritis, and hand osteoarthritis), the ‘diabetes’ variable is largely informed by those with type 2 diabetes, and soforth. Although these definitions might have resulted in misclassification of medical conditions, it is reassuring that our findings in the individual-level analyses (which did not adjust for familial confounding) generally showed associations consistent with prior work [11–14, 18, 23], arguing against differential misclassification due to self-report. Our longitudinal sample was restricted to individuals with no prior back problems diagnosed by a doctor at baseline. Since back pain is a symptom, which does not require a clinician assessment per se, clinician-diagnosed back problems may be an imperfect proxy for back pain. Additionally, we applied a different back pain definition at follow-up to assess incident CBP, which did not specify a minimum duration of pain needed to constitute ‘chronic’ or the particular location in the back where pain was experienced (thoracic or lumbar). However, given the high agreement between general back pain questions and lumbar-specific questions [51], and that thoracic pain without concurrent lumbar pain is less common [52], it is likely that our results are driven by lumbar-location pain [36]. Another limitation of this study was that the within-pair analyses conducted were limited by a small number of discordant twin pairs. Future co-twin control studies may consider evaluating these medical risk factors across multiple twin samples to produce larger samples of discordant pairs. Last, participants in the current study included male veterans only, due to the male-only composition of the VET Registry. These results may not be generalizable to women. Moreover, study participants were healthy and fit at the time of their military service two decades prior and may be more active than the general population. It is therefore unclear whether our study findings related to medical conditions- many of which are associated with physical activity levels- would extend to a more sedentary population.

## Conclusions

Arthritis, hypertension, CAD, and medical comorbidity score were associated with incident CBP in the current study. However, the association between arthritis and incident CBP was no longer present after accounting for confounding by familial factors common to both conditions. This suggests that prevention or treatment of arthritis is unlikely to be useful for prevention of CBP. Our findings cannot exclude the possibility of causal associations between CAD, hypertension, and medical comorbidities and incident CBP.

## Additional file

Additional file 1: **Table S1.** Incidence of Chronic Back Pain over 11-year Follow-up: Associations between medical conditions and incident chronic back pain, in those without physician-assessed back problems at baseline, with analyses stratified by zygosity*. (DOCX 96 kb)

## Abbreviations

CAD: Coronary artery disease
CBP: Chronic back pain
CI: Confidence interval
CSP #569: Cooperative Studies Program #569
OR: Odds ratio
SACQ: Self-Administered Comorbidity Questionnaire
US: United States
VA: Veterans Affairs
VET Registry: Vietnam Era Twin Registry

## References

[1] Lawrence RC, Felson DT, Helmick CG, Arnold LM, Choi H, Deyo RA, Gabriel S, Hirsch R, Hochberg MC, Hunder GG, Jordan JM, Katz JN, Kremers HM, Wolfe F (2008). Estimates of the prevalence of arthritis and other rheumatic conditions in the United States. Part II. Arthritis Rheum.

[2] Hoy D, March L, Brooks P, Blyth F, Woolf A, Bain C, Williams G, Smith E, Vos T, Barendregt J, Murray C, Burstein R, Buchbinder R (2014). The global burden of low back pain: estimates from the global burden of disease 2010 study. Ann Rheum Dis.

[3] Katz JN (2006). Lumbar disc disorders and low-back pain: socioeconomic factors and consequences. J Bone Joint Surg.

[4] Chou R, Shekelle P (2010). Will this patient develop persistent disabling low back pain?. Jama.

[5] Hartvigsen J, Hancock MJ, Kongsted A, Louw Q, Ferreira ML, Genevay S, et al. Lancet Low Back Pain Series Working Group.Lancet. 2018;391(10137):2356-67. 10.1016/S0140-6736(18)30480-X 29573870

[6] Livshits G, Popham M, Malkin I, Sambrook PN, Macgregor AJ, Spector T, Williams FM (2011). Lumbar disc degeneration and genetic factors are the main risk factors for low back pain in women: the UK twin spine study. Ann Rheum Dis.

[7] Suri P, Boyko EJ, Goldberg J, Forsberg CW, Jarvik JG. Association of Incident Lumbar Spine MRI findings with chronic low Back pain or radicular symptoms: a longitudinal cohort study. Submitted to BMC Musculoskeletal Disorders. 2013. 10.1186/1471-2474-15-152 PMC4024651 24886265

[8] Jarvik JG, Hollingworth W, Heagerty PJ, Haynor DR, Boyko EJ, Deyo RA (2005). Three-year incidence of low back pain in an initially asymptomatic cohort: clinical and imaging risk factors. Spine.

[9] Chou D, Samartzis D, Bellabarba C, Patel A, Luk KD, Kisser JM, Skelly AC (2011). Degenerative magnetic resonance imaging changes in patients with chronic low back pain: a systematic review. Spine (Phila Pa 1976).

[10] Croft PR, Papageorgiou AC, Thomas E, Macfarlane GJ, Silman AJ (1999). Short-term physical risk factors for new episodes of low back pain. Prospective evidence from the South Manchester Back pain study. Spine.

[11] Suri P, Pearson AM, Scherer EA, Zhao W, Lurie JD, Morgan TS, Weinstein JN (2016). Recurrence of pain after usual nonoperative Care for Symptomatic Lumbar Disk Herniation: analysis of data from the spine patient outcomes research trial. PM R.

[12] Suri P, Boyko EJ, Goldberg J, Forsberg CW, Jarvik JG (2014). Longitudinal associations between incident lumbar spine MRI findings and chronic low back pain or radicular symptoms: retrospective analysis of data from the longitudinal assessment of imaging and disability of the back (LAIDBACK). BMC Musculoskelet Disord.

[13] Koerner JD, Glaser J, Radcliff K (2015). Which variables are associated with patient-reported outcomes after discectomy? Review of SPORT disc herniation studies. Clin Orthop Relat Res.

[14] Rundell SD, Goode AP, Suri P, Heagerty PJ, Comstock BA, Friedly JL, Gold LS, Bauer Z, Avins AL, Nedeljkovic SS, Nerenz DR, Kessler L, Jarvik JG (2017). Effect of comorbid knee and hip osteoarthritis on longitudinal clinical and health care use outcomes in older adults with new visits for back pain. Arch Phys Med Rehabil.

[15] Suri P, Morgenroth DC, Kwoh CK, Bean JF, Kalichman L, Hunter DJ (2010). Low back pain and other musculoskeletal pain comorbidities in individuals with symptomatic osteoarthritis of the knee: data from the osteoarthritis initiative. Arthritis Care Res (Hoboken).

[16] Weiner DK, Fang M, Gentili A, Kochersberger G, Marcum ZA, Rossi MI, Semla TP, Shega J (2015). Deconstructing chronic low back pain in the older adult—step by step evidence and expert-based recommendations for evaluation and treatment: part I: hip osteoarthritis. Pain Med.

[17] Kauppila L.I. (2009). Atherosclerosis and Disc Degeneration/Low-Back Pain – A Systematic Review. European Journal of Vascular and Endovascular Surgery.

[18] Leino-Arjas P, Solovieva S, Kirjonen J, Reunanen A, Riihimaki H (2006). Cardiovascular risk factors and low-back pain in a long-term follow-up of industrial employees. Scand J Work Environ Health.

[19] Estublier C, Chapurlat R, Szulc P (2015). Association of severe disc degeneration with all-cause mortality and abdominal aortic calcification assessed prospectively in older men: findings of a single-center prospective study of osteoporosis in men. Arthritis Rheumatol.

[20] Kauppila Leena I., McAlindon Tim, Evans Stephen, Wilson Peter W. F., Kiel Douglas, Felson David T. (1997). Disc Degeneration/Back Pain and Calcification of the Abdominal Aorta. Spine.

[21] Kauppila LI (1995). Can low-back pain be due to lumbar-artery disease?. Lancet.

[22] Jhawar BS, Fuchs CS, Colditz GA, Stampfer MJ (2006). Cardiovascular risk factors for physician-diagnosed lumbar disc herniation. Spine J.

[23] Parreira PCS, Maher CG, Ferreira ML, Machado GC, Blyth FM, Naganathan V, Waite LM, Seibel MJ, Handelsman D, Cumming RG (2017). A longitudinal study of the influence of comorbidities and lifestyle factors on low back pain in older men. Pain.

[24] Ha IH, Lee J, Kim MR, Kim H, Shin JS (2014). The association between the history of cardiovascular diseases and chronic low back pain in south Koreans: a cross-sectional study. PLoS One.

[25] Hangai M, Kaneoka K, Kuno S, Hinotsu S, Sakane M, Mamizuka N, Sakai S, Ochiai N (2008). Factors associated with lumbar intervertebral disc degeneration in the elderly. Spine J.

[26] Schneider S, Mohnen SM, Schiltenwolf M, Rau C (2007). Comorbidity of low back pain: representative outcomes of a national health study in the Federal Republic of Germany. Eur J Pain.

[27] Heuch I, Heuch I, Hagen K, Zwart JA (2010). Associations between serum lipid levels and chronic low back pain. Epidemiology.

[28] de Luca KE, Parkinson L, Haldeman S, Byles JE, Blyth F (2017). The relationship between spinal pain and comorbidity: a cross-sectional analysis of 579 community-dwelling, older Australian women. J Manip Physiol Ther.

[29] Badley EM, Millstone DB, Perruccio AV: Back pain and co-occurring conditions: findings from a nationally representative sample. Spine (Phila Pa 1976) 2018. 10.1097/BRS.0000000000002590 29462062

[30] Goldberg, J and Fischer, M. Co-twin control methods. In: Everitt B, Howell D, editors. Encyclopedia of Statistics in Behavioral Science. Hoboken: Wiley. More information about the work is available here: https://onlinelibrary.wiley.com/doi/book/10.1002/0470013192. 2005.

[31] McGue Matt, Osler Merete, Christensen Kaare (2010). Causal Inference and Observational Research. Perspectives on Psychological Science.

[32] Eisen S, True W, Goldberg J, Henderson W, Robinette CD (1987). The Vietnam era twin (VET) registry: method of construction. Acta Genet Med Gemellol.

[33] Goldberg J, Curran B, Vitek ME, Henderson WG, Boyko EJ (2002). The Vietnam era twin registry. Twin Res.

[34] Tsai M, Mori AM, Forsberg CW, Waiss N, Sporleder JL, Smith NL, Goldberg J (2013). The Vietnam era twin registry: a quarter century of progress. Twin Res Hum Genet.

[35] Forsberg CW, Goldberg J, Sporleder J, Smith NL (2010). Determining zygosity in the Vietnam era twin registry: an update. Twin Res Hum Genet.

[36] Suri P, Boyko EJ, Smith NL, Jarvik JG, Williams FM, Jarvik GP, Goldberg J (2017). Modifiable risk factors for chronic back pain: insights using the co-twin control design. Spine J.

[37] Katz JN, Chang LC, Sangha O, Fossel AH, Bates DW (1996). Can comorbidity be measured by questionnaire rather than medical record review?. Med Care.

[38] Schisterman EF, Cole SR, Platt RW (2009). Overadjustment bias and unnecessary adjustment in epidemiologic studies. Epidemiology.

[39] Bergen SE, Gardner CO, Aggen SH, Kendler KS (2008). Socioeconomic status and social support following illicit drug use: causal pathways or common liability?. Twin Res Hum Genet.

[40] Sainani KL (2014). Explanatory versus predictive modeling. PM R.

[41] Suri P, Palmer MR, Tsepilov YA, Freidin MB, Boer CG, Yau MS, Evans DS, Gelemanovic A, Bartz TM, Nethander M, Arbeeva L, Karssen L, Neogi T, Campbell A, Mellstrom D, Ohlsson C, Marshall LM, Orwoll E, Uitterlinden AG, Rotter J, Lauc G, Psaty BM, Karlsson MK, Lane NE, Jarvik GP, Polasek O, Hochberg M, Jordan JM, van Meurs J, Jackson R, et al. Genome-wide Meta-analysis of 158,000 Individuals of European Ancestry Identifies Three Loci Associated with Chronic Back Pain. PLoS Genet. 2018; (In Press). 10.1371/journal.pgen.1007601 PMC6159857 30261039

[42] Suri P, Hunter DJ, Rainville J, Guermazi A, Katz JN (2012). Quantitative assessment of abdominal aortic calcification and associations with lumbar intervertebral disc height loss: the Framingham study. Spine J.

[43] Mantyselka P (2017). Cardiovascular risk reduction as a population strategy for preventing pain?. Scand J Pain.

[44] Pico-Espinosa OJ, Skillgate E, Tettamanti G, Lager A, Holm LW (2017). Diabetes mellitus and hyperlipidaemia as risk factors for frequent pain in the back, neck and/or shoulders/arms among adults in Stockholm 2006 to 2010 - results from the Stockholm public health cohort. Scand J Pain.

[45] Dario A, Ferreira M, Refshauge K, Harmer A, Sanchez-Romera J, Perez-Riquelme F, Cisneros L, Ordonana J, Ferreira P (2017). Mapping the association between back pain and type 2 diabetes: a cross-sectional and longitudinal study of adult Spanish twins. PLoS One.

[46] Liu CF, Lefebvre V (2015). The transcription factors SOX9 and SOX5/SOX6 cooperate genome-wide through super-enhancers to drive chondrogenesis. Nucleic Acids Res.

[47] Liu CF, Samsa WE, Zhou G, Lefebvre V (2017). Transcriptional control of chondrocyte specification and differentiation. Semin Cell Dev Biol.

[48] Smits P, Li P, Mandel J, Zhang Z, Deng JM, Behringer RR, de Crombrugghe B, Lefebvre V (2001). The transcription factors L-Sox5 and Sox6 are essential for cartilage formation. Dev Cell.

[49] Suri P, Palmer MR, Tsepilov YA, Freidin MB, Boer CG, Yau MS, Evans DS, Gelemanovic A, Bartz TM, Nethander M, Arbeeva L, Karssen L, Neogi T, Campbell A, Mellstrom D, Ohlsson C, Marshall LM, Orwoll E, Uitterlinden AG, Rotter J, Lauc G, Psaty BM, Karlsson MK, Lane NE, Jarvik GP, Polasek O, Hochberg M, Jordan JM, van Meurs J, Jackson R et al: Genome-wide meta-analysis of 158,000 individuals of European ancestry identifies three loci associated with chronic back pain. In: bioRxiv bioRxiv.

[50] Galesloot TE, van Steen K, Kiemeney LA, Janss LL, Vermeulen SH (2014). A comparison of multivariate genome-wide association methods. PLoS One.

[51] Denard PJ, Holton KF, Miller J, Fink HA, Kado DM, Marshall LM, Yoo JU (2010). Back pain, neurogenic symptoms, and physical function in relation to spondylolisthesis among elderly men. Spine J.

[52] Hartvigsen J, Nielsen J, Kyvik KO, Fejer R, Vach W, Iachine I, Leboeuf-Yde C (2009). Heritability of spinal pain and consequences of spinal pain: a comprehensive genetic epidemiologic analysis using a population-based sample of 15,328 twins ages 20-71 years. Arthritis Rheum.

