# Supplementary material for: Do medical conditions predispose to the development of chronic back pain? A longitudinal co-twin control study of middle-aged males with 11-year follow-up
Source: BMC Musculoskelet Disord. 2018 Oct 10;19:362. doi: 10.1186/s12891-018-2282-5 (PMC6178273; doi:10.1186/s12891-018-2282-5)
Supplement: Supplementary file 1 — Table S1. Incidence of Chronic Back Pain over 11-year Follow-up: Associations between medical conditions and incident chronic back pain, in those without physician-assessed back problems at baseline, with analyses stratified by zygosity*. (DOCX 96 kb) [file 12891_2018_2282_MOESM1_ESM.docx]

| **Risk Factor** | **Individual-level analysis^a^** | | **Individual-level analysis**  **(also adjusting for comorbidity score)^b^** | | **DZ-pair analysis** | | **MZ-pair analysis** | |
| --- | --- | --- | --- | --- | --- | --- | --- | --- |
|  | **OR (95% CI)** | ***p*-value** | **OR (95% CI)** | ***p*-value** |  | |  | |
| **Individual Medical Conditions** | | | | | | | | |
| Arthritis | n=2770 | | n=2745^c^ | | n=100 (50 pairs) | | n=148 (74 pairs) | |
|  | **1.8 (1.4-2.2)** | **<0.001** | **1.7 (1.3-2.2)** | **<0.001** | 1.4 (0.4-4.5) | 0.57 | 0.6 (0.2-1.7) | 0.35 |
| Diabetes | n= 2776 | | n= 2748^d^ | | N=102 (51 pairs) | | n=146 (73 pairs) | |
|  | 1.2 (0.8-1.9) | 0.33 | 1.2 (0.8-1.9) | 0.46 | 1.0 (0.2-5.0) | 0.66 | 0.3 (0.03-3.3) | 0.35 |
| Hypertension | n= 2770 | | n= 2770^d^ | | N=102 (51 pairs) | | n=146 (73 pairs) | |
|  | **1.3 (1.0-1.5)** | **0.04** | 1.2 (0.9-1.5) | 0.14 | 0.8 (0.3-2.2) | 1.0 | 2.0 (0.7-5.4) | 0.17 |
| Coronary Artery Disease (CAD) | n= 2771 | | n= 2744^d^ | | N=102 (51 pairs) | | N=146 (73 pairs) | |
|  | **1.6 (1.0-2.3)** | **0.05** | 1.5 (0.9-2.3) | 0.09 | 1.5 (0.2-9.1) | 0.60 | 3.0 (0.3-29) | 0.35 |
| **Overall Comorbidity Burden** | | | | | | | | |
| Medical comorbidity score | n=2740 | | - | | N=96 (48 pairs) | | N=144 (72 pairs) | |
|  | **1.2 (1.1-2.3)** | **<0.001** | **-** | - | 1.1 (0.8-1.4) | 0.68 | 1.2 (0.9-1.6) | 0.30 |
| Items **in bold** are statistically significant at p<0.05  Sample sizes indicate # of individuals with complete data for these variables, and within-pair analyses are restricted to pairs with complete data for all variables.  ^a^Models adjusting for age, race, education  ^b^Models adjusting for age, race, education, and comorbidity score  ^c^Model adjusting for age, race, education, and comorbidity score (arthritis not included in calculation of comorbidity score)  ^d^Model adjusting for age, race, education, and comorbidity score (diabetes, hypertension, and CAD not included in calculation of comorbidity score) | | | | | | | | |

**Table S1: Incidence of Chronic Back Pain over 11-year Follow-up:** Associations between medical conditions and incident chronic back pain, in those without physician-assessed back problems at baseline, with analyses stratified by zygosity*
